# Editorial Comment to Microscopic pulmonary tumor embolism from adenocarcinoma of the prostate

**DOI:** 10.1002/iju5.12164

**Published:** 2020-05-16

**Authors:** Taro Iguchi, Akinori Minami

**Affiliations:** ^1^ Department of Urology Osaka City University Graduate School of Medicine Osaka Japan

The article by Hattori *et al*. showed a very rare case of metastatic hormone‐sensitive prostate cancer (mHSPC) that resulted in a fatal pulmonary vascular tumor embolism.^1^ Although he had an unfortunate outcome, it will be useful for future clinical activities to share such a case.

This case had a relatively low prostate‐specific antigen (PSA) level (24.6 ng/mL) at diagnosis for the clinical stage (cT4N1M1b) and a high Gleason score of 5 + 5, which was predictive of poor prognosis. Androgen deprivation therapy (ADT) plus docetaxel or abiraterone (ABI) should be considered the standard treatment of care for this case. The choice of ADT plus ABI was reasonable in the clinical guidelines in Japan at that time. The PSA level at 1 month after the start of ADT + ABI treatment was reduced to 16.1 ng/ml (35% decline), giving the impression that the initial PSA response was poor. Matsubara *et al*. reported on the PSA kinetics in a post hoc analysis of the LATITUDE trial that looked at the therapeutic effect of ABI on 1199 patients with mHSPC, and the PSA 50% and 90% decline in ADT alone was 67% and 35%, respectively, while ADT + ABI showed very good PSA response (91% and 79%, respectively), which strongly correlated with radiological progression‐free survival (rPFS) and overall survival (OS) in patients with PSA 50% decline (hazard ratio 0.44 and 0.26, respectively).^2^ It has also been previously reported that the PSA response to ABI in metastatic castration‐resistant prostate cancer (CRPC) correlates with OS.^3^

In this case, it is difficult to judge the effect of ADT + ABI treatment from the PSA response alone, because the change in PSA after the start of ADT + ABI treatment has not been described. However, if we had been able to frequently observe the changes in PSA, we may have considered chemotherapy as a subsequent treatment.

Considering the early relapse of ADT and ABI treatment and the developed ABI‐resistant CRPC within at least 5 months, this case suggests the importance of not only PSA but also image monitoring. Now upfront hormone therapy for high‐risk mHSPC can be used for apalutamide and enzalutamide as well as ABI, but a certain number of cases have been reported to relapse very early,^4^, ^5^, ^6^ and we believe that close observation is necessary, especially early in treatment.

## Conflict of interest

Taro Iguchi has obtained research funding from Bayer and Astellas, has served as a consultant or advisor from Bayer and Astellas, has participated in the speakers’ bureau for Bayer, Janssen, Sanofi and Astellas. Akinori Minami declares no conflict of interest.
